# Investigating Differences in Health-Related Quality of Life of Greeks and Albanian Immigrants with the Generic EQ-5D Questionnaire

**DOI:** 10.1155/2013/127389

**Published:** 2013-06-24

**Authors:** Eleni Lahana, Dimitris Niakas

**Affiliations:** ^1^Nursing Department, Faculty of Nursing, Technological Educational Institute of Larissa, 411 10 Larissa, Greece; ^2^Faculty of Social Sciences, Hellenic Open University, 26335 Patras, Greece

## Abstract

*Background.* Low socioeconomic status (SES) has been related by previous studies to low self-perceived HRQoL. Health is a major determinant of the society's welfare, and few studies have determined the relevant elements that contribute to health and quality of life in Greece. *Aim.* The aim of the study was to evaluate and test for differences in HRQoL of Greek and Albanian immigrant population according to ethnicity and their demographic and SES characteristics. *Methods.* The study was conducted in a sample of 660 age-matched and gender-matched Greeks and Albanian immigrants. Moderate or severe decrease in HRQoL was assessed with the generic tool EQ-5D. Differences were statistically analyzed by *t*-test and ANOVA. Also, logistic and linear regression analyses were conducted for the dependent variables of the EQ-5D dimensions and VAS scores, respectively. *Results.* The Albanian immigrants reported better self-perceived health than their Greek counterparts. Health problems increase moderately with age and lower SES and are slightly higher for women than for men. Urbanity and superior education in both Greeks and Albaniansareassociated with worse HRQoL. *Conclusion.* There are some structural and compositional differences in the self-perceived quality of life between the two ethnicities, as estimated by EQ-5D. The combined information presents to public health providers the relevant data to assess health policies according to health needs.

## 1. Introduction

 Health-related quality of life (HRQoL) has been assessed over the years by several generic measures, and it constitutes an indirect measure of health, which without a doubt is one of the main determinants of welfare for the infra-structures of society [1]. Monitoring and evaluating health are of great importance and have proven to be beneficial from the perspective of population inequalities, unmet needs, and health promotion [2]. 

 Previous studies have addressed the association of old age and low SES (low educational level, low income, manual occupation) with lower self-perceived health [2–4], but few studies have attempted to address HRQoL differences according to ethnicity, after estimating differences according to place of residence [5]. Ethnic differences appear to affect perceived health and contribute to health inequalities, along with urbanity, since in previous studies, urban residents usually report lower HRQoL than their rural counterparts [6, 7]. A previous study, by our group [8], have reported a weak impact of ethnicity and place of residence on HRQoL as assessed by the SF-36 questionnaire but identified SES as an important and independent predictor of low HRQoL.

 HRQoL is often assessed by descriptive systems and questionnaires that involve, among others, ordinal questions on self-rated health, and many recent publications have attempted to address the impact of socioeconomic status (SES) and other social parameters on HRQoL [9]. One, among others, short and useful generic measure is the EuroQol 5D (EQ-5D), which was developed by the EuroQol study group [10] and has been translated and validated for the Greek population [11]. This questionnaire in the first part infers health states in five dimensions, and in the second part it scales overall health, both of which provide a rating of self-perceived health that can be applied in the general population, as well as in health-related economic evaluations.

 HRQoL disparities have often been attributed to differences on demographic characteristics and SES, without fully investigating other characteristics of the general Greek population like ethnicity. The aim of our study is to describe the potential differences of HRQoL according to the demographic characteristics, SES, and ethnicity, based on a dataset of Greeks and Albanians living in Thessaly, with EQ5-D. As the prevalence of rural residency and immigration has been related to low SES in a number of studies, it would be of importance to address differences in HRQoL related to SES. Therefore, taking into account that immigrants often represent a population with low HRQoL that is possibly also associated with lower SES and health inequalities, we attempted to identify such inequalities and possible differences in ethnicity and SES in Greece with the generic measure EQ5-D. 

## 2. Methods

### 2.1. Population Sample

The analysis was based on a dataset of 660 age-matched and gender-matched Greeks and Albanian immigrants of Thessaly, from a cross-sectional study that was carried out in 2006. At first, a representative sample of 1,372 individuals (18+ years old, response rate 91.4%) were selected by random sampling of building blocks and households and by random selection of one adult individual per household. The selection was proportional to the population size and institutionalized individuals, nonpermanent residents, and Albanians that did not have a fluency in Greek were excluded. The EQ-5D questionnaire was answered by voluntary face-to-face interview conducted by Greek trained personnel, and the questionnaire also incorporated questions that assessed demographic and socioeconomic characteristics (age, gender, educational level, occupation, health insurance, average monthly income, ethnicity, and place of residence). The dataset of 330 Greeks and 330 Albanians was selected from the original sample of 1,372 individuals according to age and gender and was further analyzed statistically. 

### 2.2. Variables

Our main independents variables were gender and age (18–44, 45–64 and 65+), monthly income (low (<880 Euros), medium (881–1,760), high (>1,760)) educational level (primary, superior, and university), occupation (manual, nonmanual), and place of residence (rural and urban), with the ethnicity variable categorized our sample into two groups, Greeks and Albanians, where Albanians represent the highest percentage of immigrants in Greece. The dependent variables as provided by EQ-5D were the five dimensions of the questionnaire (mobility, self-care, usual activities, pain/discomfort, and depression/anxiety) and the “visual analog scale” (VAS) presented as a thermometer from 0 to 100 rating general health. Each dimension is divided into three severity levels, corresponding to no problem, some problem, and extreme problem. If a dimension is reported with no problems, it is then considered to be at level 1, while a dimension for which there are extreme problems is included to be at level 3. 

### 2.3. Statistical Analysis

The statistical analysis was carried out with the statistical package SPSS v.16 (SPSS Inc, Chicago, IL, USA). Moderate or severe decrease in HRQoL was assessed with the generic tool EQ-5D. Differences between the subpopulation and for each dimension of the EQ-5D questionnaire were statistically analyzed by Students *t*-test and ANOVA using the demographic and SES characteristics as the dependent variables for the analysis. Logistic regression analysis with dummies was conducted for each dependent variable of the five dimensions in the questionnaire, and linear regression with dummies was performed for the dependent variable VAS. Statistical significance was set at a *P* value < 0.05.

## 3. Results 

Table 1 represents the demographic, socioeconomic, and place of residence characteristics and their distribution in the general population and in the two subpopulations, as determined by ethnicity, verifying the independent variables of our study. Student *t*-test and ANOVA analysis accordingly showed that the Greek individuals were mostly urban residents, had secondary education, and were nonmanual workers at a significant rate as opposed to the Albanians. The proportion of women in the study was higher (50.6%) in the whole sample and in both the subpopulations of Greeks and Albanians. Most of the responders were at the second age group (45–64) and their place of residence was urban, irrespective to their ethnicity. In addition, in the whole sample most of the population reported medium income (56.7%) and also the Greek population reported at a higher percentage (26.1%) low income when compared to the percentage reported by the Albanians (15.5%). Furthermore, secondary educational level was reported by 75.3% of the participants, with Greeks representing mostly the secondary and university educational levels and the Albanians the secondary educational level (86.7%). Manual occupation was reported by 69.1% of the Albanian respondents and nonmanual occupation was reported by 71.5% of the Greeks.

In Table 2 the percentages of moderate or severe problems reported by the respondents according to ethnicity in each of the EQ-5D dimensions are demonstrated, and student *t*-test and ANOVA analysis are performed with the percentage of reported moderate or severe problems for each dimension being used as the dependent variable for each of the independent predictors of the questionnaire. The Greek respondents reported more moderate/severe problems in all of the 5 dimensions of the EQ-5D in comparison to the Albanians. This increased prevalence of problems in the Greek respondents was especially evident for the dimensions of pain/discomfort and depression/anxiety, whereas the Albanians reported fewer problems in all five dimensions, with their highest percentage seen only at the depression/anxiety dimension, which was still lower than the percentage reported by the Greek subpopulation. The decreased prevalence of moderate or severe problems by the Albanians was also evident even when the subpopulations were investigated according to their demographic and SES characteristics.

 Differences though as investigated within the subpopulations showed that the Albanian men reported higher prevalence of problems in all the dimensions of EQ-5D, except for the anxiety/depression dimension, whereas Greek women reported higher prevalence of problems for the dimensions of mobility, self-care, usual activities, and depression/anxiety as opposed to Greek men.

 Health problems were reported at a higher frequency with increasing age only for the comparison between middle aged and younger aged individuals, which was more evident for the Greek subpopulation. On the other hand, for the dimension of self-care, this effect was partially inversed, since the younger Albanians were the ones that reported more problems for this dimension. Both the Greek and Albanian urban residents showed a higher prevalence of health problems in all the five dimensions. In particular, urbanity was a good predictor for the dimension of pain/discomfort in the Greek individuals and also for the dimension of depression/anxiety for both the Greek and Albanian individuals. In addition, secondary education predicted higher prevalence of problems in all the dimensions of EQ-5D irrespective to ethnicity, whereas occupation predicted higher frequency of problems in all five dimensions for Greek manual workers and for the Albanian nonmanual workers. Also, medium income was a good predictor for higher reported problems in all the EQ-5D dimensions irrespective to the Greek or Albanian ethnicity. 

 In Table 3, VAS scores for the subpopulations according to ethnicity showed that the Albanians had higher scores than the Greeks irrespective to the demographic and SES characteristics. In addition, the Albanian women, Greek men, and individuals with secondary education reported better health. Also, both Greek and Albanian rural residents have better QoL than the urban residents. Greeks with low income and Albanians with high income have better VAS scores. 

Table 4 demonstrates the logistic regression analysis that was performed in the whole sample and in the ethnicity subpopulations using the dependent variables of the EQ-5D dimensions and analyzing the independent variables with dummies. The analysis showed that Greek ethnicity always is associated with increased risks for mobility (odd ratio (OR) 0.32), self-care (OR 0.36), usual activities (OR 0.21), pain/discomfort (OR 0.17), and depression/anxiety (OR 0.33). On the other hand, men showed a slight increased risk to report problems at the anxiety dimension (OR 0.64), especially Albanian men and also Albanian men related to increased risk for problems in the dimensions of self-care, usual activities, whereas Greek women had increased risk to report problems in the pain/discomfort dimension. Also, middle age in Greeks is associated with increased risk for health problems in self-care (OR 0.22) and low income as it can be expected to increase the risk for mobility problems (OR 0.76) in the whole sample. 

Linear regressions conducted for the whole sample and also for the subpopulations according to ethnicity (Greek-Albanians) using the “VAS value” as the dependent variable are presented in Table 5. In the whole sample, the VAS-values decreased in women (as compared to men) (*B*: 3.806, *P* = 0.007), whereas after controlling for ethnicity, since the estimate was significantly lower for Greek ethnicity (*B*: − 21.116,  *P* < 0.0001 in the whole sample), VAS values still decrease for Greek women but increase for Albanian women. In addition, high income increases the VAS values scores in the whole sample but reaches statistical significance only in the Albanians with high income (*B*: 5.241, *P* = 0.003); therefore the Albanians with low income are estimated to have decreased VAS values.

## 4. Discussion

 The EQ-5D questionnaire adequately detected differences in HRQoL according to ethnicity, since the Greek population showed higher prevalence of problems in all five dimensions in comparison to the Albanians. This association was also evident by the VAS scores, since ethnicity predicted increased risk of deteriorating HRQoL in Greeks for all five dimensions. Albanian ethnicity has been previously related to better HRQoL as assessed by another generic tool, the SF-36 questionnaire, but its impact was mediocre and is counteracted by SES in this immigrant population [8]. Previous reports on the association of HRQoL according to ethnicity have identified that blacks value their health state higher than other members of the U.S. population [12], Maori value their health similarly than non-Maori in New Zealand [13], whereas in other countries immigrants report more problems with self-care and depression/anxiety and usually assess pain more dramatically [14]. It is evident therefore that ethnicity and immigrant's health states among countries may differ significantly and reference standards among immigrants might be influenced not only by the country, but also by personal or cultural beliefs about health [15] and possibly by differences in health needs. Greek immigrants of Albanian ethnicity usually come to Greece in seek for better living and often consider their immigration beneficial for their quality of life; therefore any differences could possibly be masked by their need for “survival.” Place of residence was accounted for in the sample in order to normalize any possible differences that could affect significantly our ethnicity subpopulations, and the assessment showed that such an effect was low to mediocre; therefore Greek ethnicity was a good predictor for decreased HRQoL and did not relate to urbanity. Irrespective to ethnicity though, urbanity predicted higher prevalence of problems especially for the depression/anxiety dimension.

Health inequalities according to place of residence have been associated to attainment of higher SES, and rural residents possibly rate their health higher because, as seen previously [8], they are in a more satisfactory social and natural environment, which improves their mentality [16]. Therefore, Greek and Albanian rural residents still report higher VAS scores and are not associated with low HRQoL, indicating that their general health perception is not limited by physical restrictions of environment and is mainly attributed to the differential physical and social environment and not to SES. All of the previously mentioned require further investigation and need to be addressed in other subpopulations of Greece. 

 In addition, the demographic characteristics of the population exerted similar results to previous reports [17, 18], where increasing age and women showed a higher prevalence in HRQoL mostly evident in Greek women, since Albanian women reported more problems only for the depression/anxiety dimension. Furthermore, Greek women, although were predicted to have better VAS scores, showed increased risk for all four dimensions of EQ-5D except for the pain/discomfort dimension in which only the Albanian women exhibited increased risk. 

 On the other hand, lower SES as assessed by educational level, occupation, and monthly income is associated with lower HRQoL, especially for manual occupation and low monthly income. Educational level had a low impact on HRQoL, where secondary education predicted higher prevalence for all the dimensions especially for the depression/anxiety in Greeks and Albanians. In compliance, VAS scores for education only exerted the effect of the population's characteristics and did not relate to any additive effect. Several studies have associated increased HRQoL with increased SES and have attempted to identify the impact of education, income, and occupation on self-perceived health [4, 18, 19], but most of the studies evaluate SES and HRQoL in accordance to chronic disease prevalences. The study data are in agreement with previous reports, but education still remains a mediocre predictor of lower HRQoL, which could possibly be related to cultural perception of health and might also be counteracted by the ability of attaining a better paid occupation than education itself. It is noteworthy that the Albanians showed increased HRQoL with increasing income, which was also evident by the positive estimate of the VAS score with high income, whereas manual occupation showed increased risk for health problems. In particular, manual occupation predicted more problems in Greeks, whereas nonmanual occupation predicted more problems in the Albanians, which could be possibly attributed to a better self-perception of their health status and their health needs. Previous studies have associated low SES with HRQoL inequalities mostly in the physical domain [20], whereas a study addressing the inclusion criteria in the labor market of Albanian immigrants in Greece indicated that age, gender, and ethnicity contribute significantly to the attainment of a nonmanual, well-paid and secure occupation [21]. Therefore, the HRQoL inequalities identified in Albanians according to SES can be partially attributed to ethnicity and the restrictions of social inclusion by the Greek society. 

 Some limitations in our study, such as limited number of highly educated rural residents and limited number of rural Albanians, should be taken into account. Moreover, our sample is not representative of the Greeks and Albanians living in Greece since the study was conducted in Thessaly region, Central Greece. 

## 5. Conclusions

This study demonstrates that Greek ethnicity is associated with decreased HRQoL irrespective to the demographic and SES characteristics of the population, indicating that the Albanian immigrants estimate their self-perceived health higher than their Greeks counterparts. There was a mediocre loss of HRQoL in people that are middle aged, female, with lower SES, and in an urban place of residence. It is a study that stresses the importance of elucidating health differences in Greece that provides the appropriate information for public health policy makers, in Greece as well as in Europe. At the same time, further studies investigating the association of ethnicity and place of residence in depth are still needed especially under the current Greek economic crisis. Our findings though imply that better health services and socioeconomic policies could mostly help Greek people improve their HRQoL which could be true for natives in other countries.

## Figures and Tables

**Table 1:** Demographic, socioeconomic, and place of residence characteristics of the responders in the whole sample and according to ethnicity.

	Whole sample (*n* = 660)	Greeks (*n* = 330)	Albanians (*n* = 330)
	*n* (%)	*n* (%)	*n* (%)
Gender			
Men	326 (49.4)	160 (48.5)	166 (50.3)
Women	334 (50.6)	170 (51.5)	164 (49.7)
Age			
18–44	227 (34.4)	114 (34.5)	113 (34.2)
45–64	368 (55.8)	184 (55.8)	184 (55.8)
65–75+	64 (9.7)	32 (9.7)	32 (9.7)
Place of residence		∗∗	
Rural	183 (27.7)	110 (33.3)	73 (22.1)
Urban	477 (72.3)	220 (66.7)	257 (77.9)
Monthly income			
Low	137 (20.8)	86 (26.1)	51 (15.5)
Medium	374 (56.7)	166 (50.3)	208 (63.0)
High	149 (22.6)	78 (23.6)	71 (21.5)
Education		∗	
Primary	75 (11.4)	50 (15.2)	25 (7.6)
Secondary	497 (75.3)	211 (63.9)	286 (86.7)
University	88 (13.3)	69 (20.9)	19 (5.8)
Occupation		∗∗∗	
Manual	322 (48.8)	94 (28.5)	228 (69.1)
Nonmanual	338 (51.2)	236 (71.5)	102 (30.9)
Ethnicity			
Greek	330 (50)	—	—
Albanians	330 (50)	—	—

*P < 0.05, **P = 0,001, **P < 0,0001.

**Table 2:** Percentage of Greek and Albanian people reporting moderate or severe problems in the five health-related quality of life dimensions of EQ-5D.

	Mobility		Self-care		Usual activities		Pain/discomfort		Depression/anxiety	
	Greeks	Albanians	Greeks	Albanians	Greeks	Albanians	Greeks	Albanians	Greeks	Albanians
Whole sample	34.3%	21.3%	13.0%	6.7%	35.0%	16.3%	66.7%	37.7%	65.3%	54.0%

Gender										
Men	18,5%**	11,8%	5,5%**	3,9%	18,8%*	9,4%	38,2%*	21,2%	36,4%*	24,8%
Women	24,5%**	8,8%	10,3%**	2,1%	23,9%*	6,4%	37,9%*	15,8%	38,8%*	28,5%
Age										
18–44	15,5%	7,9%	5,5%*	3,3%	15,5%	7,0%	27,3%	12,7%	24,8%	17,6%
45–64	23,6%	10,3%	9,1%*	1,5%	22,7%	7,0%	41,8%	20,9%	42,7%	30,0%
65–75+	3,9%	2,4%	1,2%*	1,2%	4,5%	1,8%	7,0%	3,3%	7,6%	5,8%
Place of residence										
Rural	14,8%*	2,7%	4,5%	0,3%	13,0%*	1,8%	24,5%	7,6%	24,8%	10,6%
Urban	28,2%*	17,9%	11,2%	5,8%	29,7%*	13,9%	51,5%	29,4%	50,3%	42,7%
Education										
Primary	6,7%	1,5%	2,4%	0,9%	6,7%	1,2%	11,2%	3,3%	12,4%	3,6%
Secondary	26,7%	17,9%	9,4%	4,8%	25,8%	12,7%	47,9%	30,9%	47,0%	45,8%
University	9,7%	1,2%	3,9%	0,3%	10,3%	1,8%	17,0%	2,7%	15,8%	3,9%
Occupation										
Manual	29,7%	5,5%	11,8%	1,8%	29,1%*	3,0%	53,0%	10,3%	53,9%*	13,9%
Nonmanual	13,3%	15,2%	3,9%	4,2%	13,6%*	12,7%	23,0%	26,7%	21,2%*	39,4%
Monthly income										
Low	13,0%	4,5%	3,9%**	2,4%	10,6%	3,6%	19,7%	7,3%	19,1%	9,4%
Medium	20,9%	12,1%	8,2%**	2,7%	22,1%	9,1%	38,8%	22,4%	38,2%	31,5%
High	9,1%	3,9%	3,6%**	0,9%	10,0%	3,0%	17,6%	7,3%	17,9%	12,4%

*P < 0.05. **P < 0.01. ***P < 0.001.

**Table 3:** Visual analogical scale (thermometer) in Greek and Albanian.

	Sample		Mean ± SD	
	Greeks	Albanians	Greeks	Albanians
Whole sample	330	330	56,25 ± 17,05	77,26 ± 18,58
Gender				
Men	160	166	60,69 ± 18,13	76,28 ± 19,88
Women	170	164	51,52 ± 14,45	78,22 ± 17,20
Age				
18–44	114	114	56,20 ± 13,79	75,49 ± 20,40
45–64	184	184	57,23 ± 18,85	79,32 ± 16,35
65–75+	32	32	50,75 ± 16,10	71,56 ± 22,59
Place of residence				
Rural	110	73	57,27 ± 18,04	79,52 ± 16,44
Urban	220	257	55,73 ± 16,56	76,61 ± 19,12
Education				
Primary	50	25	50,82 ± 18,12	72,80 ± 20,72
Secondary	211	286	57,77 ± 16,35	78,01 ± 18,10
University	69	19	55,51 ± 17,76	71,84 ± 21,93
Occupation				
Manual	94	228	56,79 ± 16,95	77,04 ± 18,27
Nonmanual	236	102	56,03 ± 17,13	77,75 ± 19,33
Monthly income				
Low	86	51	57,45 ± 16,57	69,71 ± 25,07
Medium	166	208	55,39 ± 17,85	78,08 ± 16,68
High	78	71	56,73 ± 15,91	80,28 ± 17,26

**Table 4:** Logistic regression analyses in the whole sample.

Odds ratio and 95% confidence interval															
Whole sample with pseudovariables															
Independent variables	Mobility			Self-care			Usual activities			Pain/discomfort			Depression/anxiety		
	W.S.	Gr.	Alb	W.S.	Gr.	Alb	W.S.	Gr.	Alb	W.S.	Gr.	Alb	W.S.	Gr.	Alb
Gender (men versus women)	0,82	1,50	0,54	0,74	2,44	0,42**	0,87	1,74	0,59**	1,20	1,61*	0,82	0,64**	0,72	0,55**
	0,581–1,166	0,855–2,622	0,340–0,847	0,442–1,239	0,874–6,799	0,222–0,796	0,608–1,257	0,923–3,263	0,376–0,934	0,847–1,689	1,010–2,562	0,488–1,392	0,459–0,906	0,459–1,133	0,321–0,927

Age (middle versus younger)	1,11	0,75	1,09	1,65	0,22*	1,38	1,32	0,57	0,91	1,08	1,00	0,90	1,12	1,16	1,36
	0,747–1,653	0,425–1,307	0,685–1,738	0,896–3,029	0,073–0,674	0,733–2,604	0,871–2,012	0,302–1,064	0,573–1,448	0,721–1,621	0,622–1,600	0,529–1,545	0,755–1,658	0,732–1,829	0,802–2,318

Place of residence (urban versus rural)	0,93	1,91	0,90	0,83	6,44	1,32	0,77	1,47	1,19	0,97	0,92	1,19	1,22	1,00	1,04
	0,656–1,330	0,803–4,522	0,560–1,454	0,494–1,385	0,733–1,479	0,677–2,577	0,533–1,112	0,525–4,122	0,735–1,916	0,680–1,370	0,481–1,752	0,692–2,036	0,866–1,723	0,539–1,847	0,604–1,784

Education (secondary versus primary)	1,14	1,12	1,11	1,10	0,86	1,10	1,25	1,84	1,11	1,24	1,18	1,26	0,96	1,38	0,79
	0,813–1,609	0,526–2,369	0,752–1,635	0,679–1,768	0,256–2,866	0,658–1,855	0,877–1,769	0,799–4,245	0,751–1,630	0,859–1,778	0,622–2,235	0,803–1,977	0,674–1,379	0,734–2,603	0,504–1,232

Occupation (manual versus nonmanual)	0,81	1,09	0,78	1,23	2,48	1,24	0,69	0,68	0,72	0,79	0,82	0,68	0,77	0,61	1,02
	0,552–1,199	0,550–2,168	0,476–1,278	0,696–2,183	0,793–7,746	0,618–2,477	0,456–1,030	0,293–1,566	0,441–1,179	0,535–1,178	0,464–1,453	0,375–1,240	0,523–1,126	0,357–1,058	0,581–1,791

Monthly income (high versus low)	0,76*	0,78	0,78	0,81	0,55	0,98	0,92	0,74	1,04	0,84	0,76	0,93	1,02	0,92	1,12
	0,580–0,989	0,493–1,242	0,560–1,094	0,551–1,183	0,246–1,211	0,625–1,526	0,700–1,212	0,444–1,234	0,747–1,452	0,641–1,098	0,512–1,119	0,635–1,370	0,784–1,328	0,634–1,346	0,765–1,640

Ethnicity (Greeks versus Albanians)	0,32***			0,36***			0,21***			0,17***			0,33***		
	0,217–0,470			0,200–0,663			0,136–0,315			0,116–0,249			0,226–0,477		

*P < 0.05, **P < 0.01, ***P < 0.001.

Whole sample (W.S.), Greeks (Gr.), and Albanians (Alb).

**Table 5:** Vas evaluation of health states according to demographic, socioeconomic, place of residence, and ethnicity multivariate linear regression analyses.

Independent variables	Whole sample		Greeks		Albanians	
	*B*	*P* value	*B*	*P* value	*B*	*P* value
Gender (men versus women)	3.806	0.007	9.902	0.000	−1.959	0.346
Age (older versus younger)	−0.653	0.584	−2.412	0.132	1.457	0.401
Place of residence (urban versus rural)	−2.279	0.159	−0.912	0.638	−2.013	0.481
Occupation (manual versus nonmanual)	−0.378	0.811	−0.334	0.869	−0.591	0.814
Monthly income (high versus low)	1.544	0.160	−1.489	0.271	5.241	0.003
Ethnicity (Greeks versus Albanians)	−21.116	0.000				
*R* ^2^	0.272		0.089		0.033	
